# Supplementary material for: Slow-growing Salmonella enterica serovariety Typhi mis-identified as serovariety Gallinarum in Ibadan, Nigeria
Source: PLOS Glob Public Health. 2025 Jul 7;5(7):e0004745. doi: 10.1371/journal.pgph.0004745 (PMC12233226; doi:10.1371/journal.pgph.0004745)
Supplement: Attachment — Submitted filename: Response to editorial review of PGPHv2.docx [file pgph.0004745.s003.docx]

Response to editorial review of PGPH-D-25-00257

Request made on April 4

Use of isolates obtained in national laboratory-based surveillance for blood borne isolates at sentinels in Ibadan and elsewhere is covered by approval of Proposal UI/EC/21/0191. Approval of proposal UI/EC/22/0113 provides permission for use of surveillance isoaltes from all sentinels in Nigeria, including Ibadan for research. Lines 60-65 contain this expanded information in the revised manuscript

The specific dates when the isolates used were collected are now listed in full on 75-76 and 77-80 in response to the editorial query.

Request made on Feb 17^th^

Before we can proceed, please report in the Methods section the day, month and year of the start and end of the recruitment period for this study.

This study came out of Salmonella surveillance that began in 2017 and continues until this day. The specific isolates for this study were obtained in 2021 and 2022. This is now mentioned in the manuscript on lines 70-72 and ethics approvals covering that period have been uploaded.

Requests made on Feb 6

1. Please ensure that the Author List in your manuscript file matches the Author List in the online submission form, including initials and order. Currently, author "Oluwafemi Popoola" name listed in manuscript file is different from online submission form.

Please use either initials or full names in both the manuscript and online submission form.

The submission was redone using the same names on the submission system and in the manuscript

2. Please complete all items on the Human Participants Research Checklist that are relevant for your submission, by following this link: <http://journals.plos.org/plosone/s/file?id=dc11/PLOSOne_Human_Subjects_Research_Checklist.docx>. There may be overlap between the checklist items and other queries listed here; please address any duplicated queries both in your response email and on the checklist itself. Upload the completed Human Participants Research checklist as file type “Other” when you re-submit your manuscript. This document is for internal journal use only and will not be published if your article is accepted. The requested information will help us to assess whether your submission complies with PLOS’s policies and adheres to applicable reporting standards. Note that your manuscript may be rejected if you provide incomplete or inadequate responses to the checklist questions and that changing the ‘Section/Category’ of your article does not affect this requirement.

The human participants research checklist was completed and uploaded.
