# Supplementary material for: Slow-growing Salmonella enterica serovariety Typhi mis-identified as serovariety Gallinarum in Ibadan, Nigeria
Source: PLOS Glob Public Health. 2025 Jul 7;5(7):e0004745. doi: 10.1371/journal.pgph.0004745 (PMC12233226; doi:10.1371/journal.pgph.0004745)
Supplement: Attachment — Submitted filename: Response to reviewer comments_GTS_PO_INOv2.docx [file pgph.0004745.s004.docx]

**Reviewer #1:** This well-designed study documents three S. Typhi bloodstream isolates that were erroneously called S. Gallinarum in a routine clinical laboratory, then re-typed by biochemical retesting, Illumina + Nanopore sequencing, and genomic analysis. The work has clear public-health relevance for typhoid-endemic, resource-limited settings and illustrates how rrn-mediated chromosomal rearrangements can generate slow-growing S. Typhi with atypical phenotypes.

**Major comments**
1. Figure 4 has poor resolution. Please supply individual high-resolution (≥300 dpi) TIFF/PNG files. It is impossible to read the maps.

**Author’s response:** We have reconstructed Fig 4 at much higher resolution in response to the reviewer’s comment

2. The Discussion would benefit from limitations paragraph acknowledging (i) the very small number of cases and (ii) potential geographic specificity; please consider other limitations.

**Author’s response:** We have expanded the content of the discussion thoroughly to include limitations in response to the reviewer’s comment on lines 361-368.

3. Only a single p=0.001 Fisher test is reported (GGT prevalence). Please specify software (e.g., R v4.3), exact two-sided/one-sided test, and numerator/denominator to aid reproducibility.

**Author’s response:** The Fisher’s exact test performed was two-sided and conducted with EpiInfo. This has been added to the manuscript on lines 189-90. Strain numbers in categories have been added to the results section on lines 225-226 so that the computation can be repeated, as requested by the reviewer.

**Minor comments:**
line 47: import -> importance, line 47, 67 and others.... Salmonella should be italic

**Author’s response:** We have implemented this in lines 47, 87 and throughout the paper

Reviewer #2: The authors presented an interesting case of using NGS to correctly detect slow-growing serovar Typhi that were previously misidentified as serovar Gallinarum. There are a few comments that I would recommend to the authors to consider:

1. The Biosamples of the 3 isolates do not exist on NCBI. The authors should look into making this rectified

**Author’s response:** The release date of the biosamples was originally scheduled for a later time but has since been made public. (<https://www.ncbi.nlm.nih.gov/biosample/45804238>, <https://www.ncbi.nlm.nih.gov/biosample/45804239>, <https://www.ncbi.nlm.nih.gov/biosample/45804240>)

2. In the abstract, the first mention of S. Gallinarum should be Salmonella enetrica subsp. enterica Gallinarum. Also it shouldn't be abbreviated as S. Gallinarum but preferably as serovar Gallinarum given that is a serovar and not a species name of Salmonella. Kindly correct throughout the manuscript. The same applies to S. Typhi. It should be corrected to serovar Typhi.

**Author’s response:** Thank you for your comment regarding the nomenclature of *Salmonella enterica* subsp. *enterica* serovars in the abstract and throughout the manuscript. As suggested, we have amended the first mention in the abstract to the full designation "*Salmonella enterica* subsp. *enterica* serovar Gallinarum" and made a similar correction for *Salmonella enterica* subsp. *enterica* serovar Typhi

on lines 17-18, 49 and 64.

However, for subsequent mentions, we have used the widely accepted and conventional abbreviation format "***S.* Gallinarum**" and "***S.* Typhi**", in which the genus is abbreviated and italicized, and the serovar is capitalized and non-italicized. This format aligns with established nomenclature guidelines from the WHO Collaborating Centre for Reference and Research on Salmonella (<https://www.pasteur.fr/sites/default/files/veng_0.pdf>) and is common practice in microbiological literature (<https://www.woah.org/app/uploads/2021/05/salmonella-enterica-all-serovarsinfection-with.pdf> ). Accordingly, this convention has been implemented consistently throughout the manuscript.

3. The introduction could use some historical context and more information on the different serovars of S. enterica and their public health implications. The Gallinarum serovar is usually associated with poultry and the authors should also address its risk as a zoonotic pathogen

**Author’s response:** We have revamped and expanded the scope and content of the introduction thoroughly, on lines 53-76 in response to the reviewer’s comment.

5. Line 171 Gallinarum not GAllinarum

**Author’s response:** We have corrected this in line 191.

6. Figure 1, what does INTS signify? The figure caption should be self explanatory without reading the article

**Author’s response:** We have edited this in figure 1 so that it is clear that iNTS are “invasive non-typhoidal Salmonella”

7. Figure 4 is hard to read a more legible Figure would be appropriate

**Author’s response:**

We have provided individual high-resolution graphics of each chromosomal rearrangement. To accommodate the need for a grouped view, we have also attached a separate PNG and SVG file that combines all rearrangements into one image.
