# Supplementary material for: Slow-growing Salmonella enterica serovariety Typhi mis-identified as serovariety Gallinarum in Ibadan, Nigeria
Source: PLOS Glob Public Health. 2025 Jul 7;5(7):e0004745. doi: 10.1371/journal.pgph.0004745 (PMC12233226; doi:10.1371/journal.pgph.0004745)
Supplement: Attachment — Submitted filename: Response to Reviewers.docx [file pgph.0004745.s005.docx]

Please find below a list of requested changes that were made to improve the manuscript

Journal Requirements:

Please review your reference list to ensure that it is complete and correct. If you have cited papers that have been retracted, please include the rationale for doing so in the manuscript text, or remove these references and replace them with relevant current references. Any changes to the reference list should be mentioned in the rebuttal letter that accompanies your revised manuscript. If you need to cite a retracted article, indicate the article’s retracted status in the References list and also include a citation and full reference for the retraction notice.

**Author response:** *We have replaced the previous reference "9" with reference "1" as they are identical. We reviewed all references and confirm that none have been retracted as at 30 May 2025. (That is, we found no retraction notifications for any of the references.) All articles are from NCBI-indexed journals except for two: reference 10 (https://www.academia.edu/download/58422112/Diseases_of_Poultry_Y_M_saif.pdf#page=645) and reference 13 (https://www.researchgate.net/publication/215806562_Salmonella_gallinarum_septicaemia_in_humans).*

*References 10 and 13 were added per the reviewer's request for reports of Salmonella Gallinarum infections in humans.*

Additional Editor Comments
Thank you for your revised manuscript submission. However, the track-changed version provided does not show all tracked changes. Could you please resubmit a version of the manuscript with all changes clearly marked using Word's Track Changes feature?

**Author response:** *We apologise if there were changes not marked up. We have used the Compare Tool in Word to generate a completely marked up version without error.*

The figures provided are still not sufficiently clear for publication. We kindly request that you submit improved versions of all figures with higher resolution and enhanced clarity.

**Author response:** We have used PACE to review and adjust the images so that they meet the journal’s requirements. Prior to PACE review, we also created a bolder version of Fig 4, which is the figure reviewers were most concerned about.
